# ICF syndrome: An epigenetic paradigm for primary immunodeficiencies

**DOI:** 10.70962/jhi.20250165

**Published:** 2025-12-05

**Authors:** Ricardo Martins-Ferreira, Esteban Ballestar

**Affiliations:** 1 Epigenetics and Immune Disease Group, Josep Carreras Leukaemia Research Institute, Barcelona, Spain; 2 Epigenetics in Inflammatory and Metabolic Diseases Laboratory, Health Science Center, East China Normal University, Shanghai, China

## Abstract

Immunodeficiency, centromeric instability, and facial anomalies (ICF) syndrome is a rare autosomal recessive disorder and a paradigmatic epigenetic inborn error of immunity. It is caused by mutations in genes essential for DNA methylation and chromatin regulation - DNMT3B (ICF1), ZBTB24 (ICF2), CDCA7 (ICF3), and HELLS (ICF4). All subtypes share hypogammaglobulinemia, centromeric instability of chromosomes 1, 9, and 16, and characteristic facial anomalies. ICF highlights the role of DNA methylation in genome stability and immune development: DNMT3B deficiency impairs de novo methylation, while the ZBTB24–CDCA7–HELLS axis affects heterochromatin remodeling and methylation maintenance. These defects drive chromosomal abnormalities and immune dysfunction, including impaired B cell maturation and class-switch recombination. Although each subtype shows distinct genotype–phenotype correlations, all converge on altered methylation of repetitive DNA and deregulated immune-related genes. ICF serves as a key model to understand how epigenetic disruption causes immunodeficiency. Limited treatments include supportive care and hematopoietic stem cell transplantation. Insights from ICF also inform other primary immunodeficiencies with epigenetic alterations, such as common variable immunodeficiency.

## Teaser

Martins-Ferreira and Ballestar review how ICF syndrome serves as a paradigm of epigenetic immunodeficiency. They show how alterations in DNA methylation and chromatin remodeling disrupt genome stability and B cell maturation, highlighting broader principles linking epigenetic regulation to immunity and informing other primary immunodeficiencies.

## Introduction

The immunodeficiency, centromeric instability, and facial anomalies (ICF) syndrome is a rare autosomal recessive inborn error of immunity (IEIs). It was first described in 1978 in two patients with specific facial features, immunodeficiency, and instability of pericentromeric regions of chromosomes 1, 9, and 16 (1, 2, 3). The current number of patients described is estimated to be over 118 (4), displaying genetic heterogeneity, variable immunodeficiency, mild facial dysmorphism, and centromeric chromosomal instability. Symptoms commonly appear early in childhood, and life expectancy is severely decreased if left untreated (5).

The immunodeficiency phenotype centers predominantly on B cells and includes hypo- or agammaglobulinemia. Patients often exhibit normal B cell counts but show impairments in the late stages of B cell differentiation. A lack of memory B cells and an increased proportion of naïve B cells have been shown, along with potential autoreactivity and increased apoptosis upon activation (6). Mechanistically, this is considered to be associated with defective isotype switching of immunoglobulins, although discordant behaviors can be observed between patient subclasses, as will be discussed in detail below. Regarding T cells, counts are commonly within normal ranges, although a small fraction of cases have shown decreased numbers of CD4^+^ cells, and impaired proliferation upon stimulation. A tendency toward T cell count deficiency is more frequently observed in older children and young adults (5, 7, 8). In a more recent T cell–directed study, ICF patients showed overall decreased CD4^+^ T cells, and defective proliferation was also observed in CD8^+^ T cells (9). There is a new hypothesis regarding potential implications of chromosome instability and hypomethylation in T cell differentiation in ICF syndrome. However, due to the lack of existing research on this subject, it will not be covered in this review. Infections are prominent among patients and represent the leading cause of death, particularly respiratory infections, but also those affecting the gastrointestinal tract and cases of sepsis (5, 7). Given the prominence of the immunodeficiency phenotype in these patients, treatment commonly consists of intravenous immunoglobulin (IVIG) replacement, and in some severe cases, hematopoietic stem cell transplantation (HSCT) (5, 7, 10), both of which result in favorable clinical and immunological outcome (11).

The cytogenetic hallmark of ICF is centromeric instability, particularly affecting chromosomes 1, 9, and 16, and is associated with CpG hypomethylation of the pericentromeric satellite II and III repeats. Decondensation of these regions makes them susceptible to breakage and rejoining, leading to multibranched configurations. These patterns were initially described as predominant in lymphoid cells but are not restricted to the hematopoietic cell lineage (3, 12). Hypomethylation has also occasionally been demonstrated in α-satellite DNA, as well as in non-satellite regions such as Alu, *D4Z4* and *NBL2* repeats, the *PGK1* gene and other X-linked sequences, *H19* and other imprinted genes (13). Additional chromosomal abnormalities associated with ICF syndrome include short telomeres with RNA:DNA hybrids, which are linked to high levels of chromosomal damage (14, 15).

The vast majority of ICF patients present with facial anomalies, with epicanthic folds, hypertelorism, flat nasal bridge, and low-set ears being the most common features. Although not life-threatening, these features may serve as diagnostic clues. Additional clinical manifestations may include autoimmunity, growth retardation—possibly associated with low birth weight—delays in cognitive, speech, and gross motor development, as well as congenital defects such as inguinal hernia, hypospadias, cleft palate, syndactyly, and cerebral malformations.

Moreover, a predisposition to cancer may also be observed in ICF patients, with reports of angiosarcoma, acute lymphoblastic leukemia, and Hodgkin lymphoma. However, the overall cancer prevalence in ICF patients is low (5, 7, 10). *Dnm3b-*mutant mice also show higher predisposition to tumorigenicity, which may be mechanistically attributed to chromosomal instability and impaired immune surveillance (16).

Despite the current understanding of the diverse pathomechanisms implicated in the development of ICF syndrome, the limited number of characterized patients leaves considerable uncertainty in the field. There is still no consensus on how chromatin defects and locus-specific methylome alterations relate to the full spectrum of clinical manifestations, why some of these features are not shared by all patients, or how genotype correlates with phenotype. Notwithstanding these limitations, the current knowledge on the genetics and epigenetics of ICF—and their possible links to immunodeficiency and other IEIs—is discussed in the sections below.

## Genetics of ICF: Mutations in epigenetic and chromatin factors

**Figure 1.**
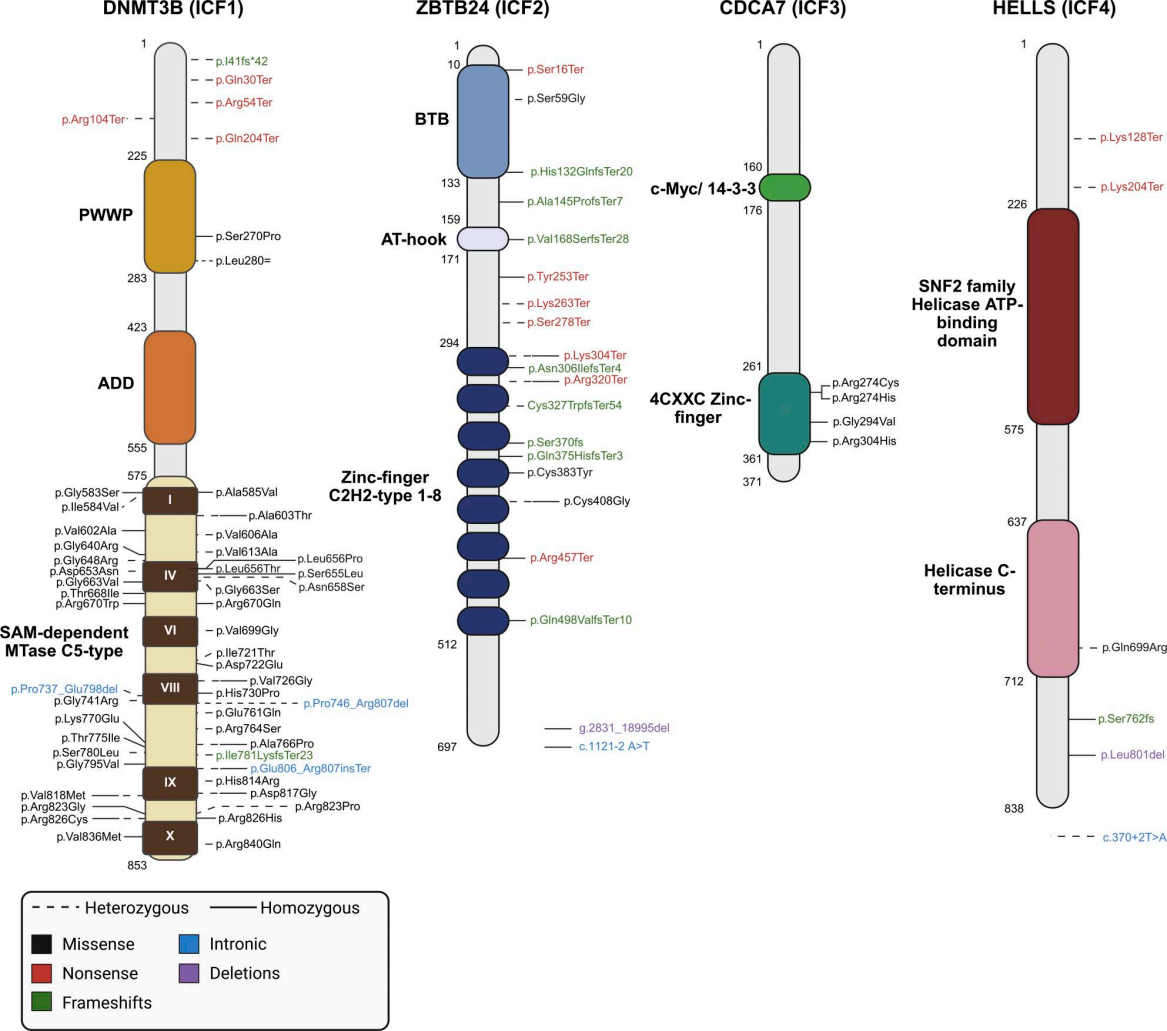
**A resource of mutations identified in ICF syndrome.** Schematic comprehensive representation of the mutations described in ICF syndrome patients, mapped to the protein sequence, for DNMT3B or ICF1 (genomic location: chr20; protein isoform: NP_008823.1), ZBTB24 or ICF2 (genomic location: chr6; protein isoform: NP_055612.1), CDCA7 or ICF3 (genomic location: chr2; protein isoform: NP_665809.1), and HELLS or ICF4 (genomic location: chr10; protein isoform: NP_060533.2). Information on mutations, protein structures, and their locations was collected from ClinVar (20), UniProt (21, 22), InterPro (22), icn3d (23), and Atlas of Genetics and Cytogenetics in Oncology and Haematology (24), and the respective reference studies (DNMT3B [5, 7, 10, 11, 25, 26, 27, 28, 29, 30, 31, 32, 33, 34, 35, 36, 37, 38, 39, 40, 41, 42, 43, 44, 45, 46], ZBTB24 [7, 8, 11, 17, 32, 33, 47, 48, 49, 50, 51, 52, 53, 54, 55, 56, 57, 58, 59], CDCA7 [18], and HELLS [18, 30, 60]). Missense mutations are represented in black, nonsense mutations in red, frameshifts in green, intron mutations in blue, and deletions in purple. Dashed lines represent mutations identified in heterozygosity, and solid lines mutations in homozygosity. Some mutations have been described in both heterozygosity and homozygosity in different patients, e.g., p.Ala603Thr in DNMT3B. The genetic variant notations used were collected from ClinVar and from the original studies, if a ClinVar entry was non-existing; furthermore, the notations from CDCA7 and HELLS were collected exclusively from (18). DNMT3B harbors the following annotated domains: ATRX-Dnmt3-Dnmt3L (ADD) and PWWP at N terminus that mediate chromatin reading and binding through interactions with histone modifications, and the C-terminal catalytic domain SAM-dependent MTase C5-type, which includes six conserved motifs (I, IV, VI, VIII, IX, and X). ZBTB24 is composed at N terminus by a BTB domain, involved in oligomerization and/or dimerization, and AT-hook domain, which binds to AT-rich DNA sequences, as well as eight C2H2-type zinc-finger domains in the C terminus that mediate the transcription factor function through sequence-specific DNA recognition. CDCA7 interacts with MYC and 14-3-3 proteins via a specific domain at its N terminus and the C-terminal zinc-finger domain (4CXXC) is required for recognition of DNA sequences rich in hemimethylated CpGs. HELLS possesses an N-terminal SNF2 family helicase ATP-binding, an ATPase domain responsible for mediation of nucleosome sliding and chromatin remodeling, and a C-terminal helicase motif.

ICF is a genetically heterogeneous disease, with pathogenic variants identified in four genes. Genetic studies performed on the first described patients led to the identification of mutations in *DNA methyltransferase (DNMT) 3B* (*DNMT3B*) (Box 1), defining what is now known as ICF type 1 syndrome (ICF1). Additional pathogenic genes were only identified in the 2010s: *Zinc-finger and bric-à-brac, tramtrack, broad complex (BTB) domain-containing 24* (*ZBTB24*, ICF2) (17), and a few years later, *cell division cycle associated 7* (*CDCA7*, ICF3) and *helicase lymphoid specific* (*HELLS*, ICF4) (18). It is estimated that ICF1 accounts for ∼50% of cases, ICF2 for 30%, and ICF3 and ICF4 for the remaining patients (5, 7, 8, 17, 18). A small fraction of patients—historically referred to as ICFX—lack mutations in these four genes—suggesting the involvement of undiscovered genes or atypical cases. Notably, a mutation in *ubiquitin-like with PHD and RING finger domains 1* (*UHRF1*) (DNMT1-accessory factor) was reported in a patient with an “ICF-like” phenotype (19). Given this, one could speculate that other chromatin remodeling genes could be implicated in ICF, in ongoing sequencing studies in undiagnosed cases. Fig. 1 presents a comprehensive compilation of the mutations in ICF patients, collected from ClinVar (20), UniProt (21, 22), InterPro (22), icn3d (23), and the Atlas of Genetics and Cytogenetics in Oncology and Haematology (24), as well as from the respective reference studies (DNMT3B [5, 7, 10, 11, 25, 26, 27, 28, 29, 30, 31, 32, 33, 34, 35, 36, 37, 38, 39, 40, 41, 42, 43, 44, 45, 46], ZBTB24 [7, 8, 11, 17, 32, 33, 47, 48, 49, 50, 51, 52, 53, 54, 55, 56, 57, 58, 59], CDCA7 [18], and HELLS [18, 30, 60]).

Box 1 DNMTs and epigenetic control

Mammalian DNMTs comprise a family of enzymes responsible for adding methyl groups to cytosine residues, particularly at CpG dinucleotides. DNMT3A and DNMT3B carry out de novo methylation, establishing new methylation marks in the DNA, while DNMT1 maintains these patterns during DNA replication associated with cell division. DNMT3L is a regulatory cofactor that enhances de novo methylation, whereas the ubiquitin-protein ligase UHRF1 recruits DNMT1 to hemimethylated regions. Collectively, these enzymes control gene expression, preserve genome integrity, and play indispensable roles in development and cellular identity (61).

The counterparts to the acquisition of DNA methylation are demethylation mechanisms. DNA demethylation can occur passively during DNA replication or actively through Ten-Eleven translocation (TET) enzymes, which iteratively oxidize 5-methylcytosine (5mC) to 5-hydroxymethylcytosine (5hmC), 5-formylcytosine (5fC), and 5-carboxylcytosine (5caC). Each of these oxidized forms can be diluted during DNA replication or, in the case of 5fc and 5caC, excised by thymine DNA glycosylase followed by base excision repair, resulting in replacement with unmethylated cytosines (62).

DNMTs and transcription factors can interact bidirectionally: the methylated state of regulatory CpG sites (or CpG islands) can influence the binding of transcription factors essential for the acquisition of specific cellular programs, such as those critical in B cell differentiation. In addition, certain transcription factors mediate the recruitment of DNMTs (or TET enzymes) and therefore directly influence changes in DNA methylation in a sequence-specific manner. These factors, either upstream or downstream of DNMT function, may represent important targets for understanding immunodeficiencies associated with epigenetic dysregulation.

DNMT3B is one of two de novo DNMTs (along with DNMT3A) responsible for establishing DNA methylation patterns during embryogenesis and hematopoietic differentiation, processes essential for gene regulation and genomic stability (63, 64, 65). DNMT3B specifically targets repetitive DNA elements, including pericentromeric satellite repeats and gene-regulatory regions, which require high steady-state levels of DNA methylation to maintain proper genome structure and stability (66, 67). It is essential for development, as knockout mice do not survive (68). This aligns with observations in ICF1 patients, where loss-of-function nonsense mutations at the N terminus are typically found in compound heterozygosity with missense mutations, which are predominantly located at the C-terminal methyltransferase domain. The DNMT3B protein also contains chromatin reading ATRX-Dnmt3-Dnmt3L (ADD) and Pro-Trp-Trp-Pro (PWWP) domains, which mediate recruitment to the genome through interactions with histone modifications. A homozygous pathogenic variant has been identified at PWWP (p.Ser270Pro); however, it has also been shown that DNMT3B is recruited to heterochromatin regions in a PWWP-independent manner (69) (Fig. 1). Throughout the years, many of these mutations have been shown to functionally impair DNMT3B. These include p.His814Arg, p.Asp817Gly, and p.Val818Met, which disrupt the assembly of the homodimer, and p.Arg823Gly, which reduces DNA-binding affinity and causes a shift in the sequence preference (38). Moreover, p.Leu656Pro, p.Leu656Thr, and p.Arg670Gln have also been shown to negatively affect the assembly of the DNMT3B–DNMT3B complex, coupled with lower DNA-binding affinity, differential binding to pericentric heterochromatin regions, and generally reduced global methylation (70).

The ZBTB24 protein harbors a BTB domain and AT-hook DNA-binding domain in its N-terminal region, as well as eight C2H2-type zinc-finger domains in the C terminus (Fig. 1). The specific functions and targets of ZBTB24 remain unknown; however, it belongs to a family of transcription factors involved in B cell differentiation and malignancy and is capable of recognizing methylated DNA through its zinc fingers (e.g., BCL-6, ZBTB33, or Kaiso) (71, 72). In contrast to DNMT3B, most ICF2 mutations are nonsense or frameshift variants (many of which are homozygous) (Fig. 1), resulting in premature stop codons and truncated inactive proteins. These findings indicate that the complete loss of ZBTB24 function is compatible with human life, unlike what has been reported in mice (73). Mutations in the zinc-finger domains have been shown to impair the ability of ZBTB24 localization to heterochromatic regions and may mediate DNA methylation in a manner independent of direct MTase activity (52). ZBTB24 is ubiquitously expressed, with the highest levels observed in naïve B cells, and appears to be coregulated with *DNMT3B* during B cell differentiation, suggesting its association with impaired negative B cell selection and defective late-stage B cell development observed in ICF syndrome (17).

CDCA7 is a MYC- and Notch-regulated nuclear protein shown to be involved in the emergence of hematopoietic stem cells (74, 75). It partners with HELLS, a SNF2 ATPase family protein crucial for the regulation of DNA methylation (76), forming a complex responsible for structuring pericentromeric heterochromatin. All mutations described in CDCA7 are homozygous missense variants located in the conserved C-terminal 4CXXC zinc-finger domain (18) (Fig. 1), which is responsible for the repressive activity in the homologous transcriptional repressor *CDCA7-like* (*CDCA7L*). By homology, these ICF3 mutations may disrupt the repressive effect of CDCA7 (77).

Mutations described in *HELLS* include a heterozygous missense mutation in the conserved helicase domain, an intronic mutation that leads to the destruction of the splice donor site in intron 5, a homozygous frame-shift resulting in a premature stop codon in exon 20, a homozygous deletion at C-terminal, and a compound heterozygous nonsense mutation along with a duplication that introduces a stop codon at the N terminus (18, 60) (Fig. 1). These findings suggest that the complete deficit of HELLS is compatible with human life, contrary to observations in mice (78).

The main molecular modification shared by all ICF patients is the loss of methylation in pericentromeric regions. This normally compact and cohesive heterochromatin becomes loosened, allowing aberrant recombination between repetitive sequences on different chromosomes, leading to breakage and multibranched conformations. These structural abnormalities manifest as impaired lymphocyte development and contribute to the broader clinical phenotype. Common links have been identified among all the four ICF-associated genes, such as the recruitment and genomic co-localization of DNMT3B by ZBTB24 (79, 80), and the coordinated mechanisms involving the ZBTB24–CDCA7–HELLS axis (81). The prevailing hypothesis is that DNMT3B is primarily involved in the establishment of centromeric CpG methylation, whereas ZBTB24, CDCA7, and HELLS work cooperatively to maintain methylation at these repetitive sequences. The complete landscape of the mechanisms underlying these epigenetic alterations is explored in the next section.

## Epigenetic mechanisms in ICF

DNA methylation is a major epigenetic mechanism involved in gene silencing and genomic stability. It occurs predominantly at the 5′ position of cytosine residues (5 mC) within CpG dinucleotides and is catalyzed by DNMTs (Box 1). DNA methylation patterns inherited from the parental gametes are largely erased during early embryogenesis, followed by a wave of de novo methylation that remodels the entire genome. This is then fine-tuned through coordinated adaptation during lineage commitment and differentiation (82).

In mammals, CpG sites are abundant in satellite DNA, repetitive elements, and non-repetitive intergenic regions. These sequences are typically methylated, thereby repressing their expression and preventing their expansion (83). The heterochromatic packaging of these repetitive sequences plays a key role in shaping genomic architecture and maintaining stability (84).

In mouse embryonic stem cells (mESCs), Dnmt3B has been shown to be uniquely required for methylation of pericentromeric repeats. *Dnmt3b* knockout embryos display an almost complete loss of methylation in these regions, whereas *Dnmt3a* knockout does not lead to major changes (68). In lymphoblastoid cell lines (LCLs) from ICF patients, DNMT3B dysfunction has been associated with aberrant expression of numerous genes whose regulatory regions show altered methylation. These changes are often accompanied by alterations in histone marks and include genes involved in germline function, neurogenesis, and immune responses (e.g., *CD27*, *PTPRC* [CD45]) (85, 86).

Moreover, most transcriptomic changes caused by DNMT3B mutations are likely due to trans effects, as promoter methylation of deregulated genes typically shows no significant differences in patients. These effects may result from altered nuclear organization and replication timing due to chromosome instability and may involve global changes in histone modifications, displacement of regulatory complexes, such as polycomb repressive complex 2 and loss of heterochromatin compaction (85, 87).

The molecular mechanisms underlying mutations in *ZBTB24*, *CDCA7*, and *HELLS* in ICF are not entirely straightforward but have become increasingly clarified in recent years. Despite challenges in defining the specific functions for ZBTB24, it has been proposed to act as a transcriptional activator and as a coordinator of genome-wide DNA methylation (79, 80). Its deficiency has been associated with the overexpression of multiple genes, including those involved in immune responses. In addition, ZBTB24 is located at centromeric repeat sequences and contributes to both the maintenance and establishment of DNA methylation in these regions. This is thought to occur via the recruitment of DNMT3B. Notwithstanding these findings, the precise role of ZBTB24 in regulating DNMT3B activity remains to be fully elucidated, as the existence of a direct interaction has been questioned and is likely to be indirect (18, 80, 88).

**Figure 2.**
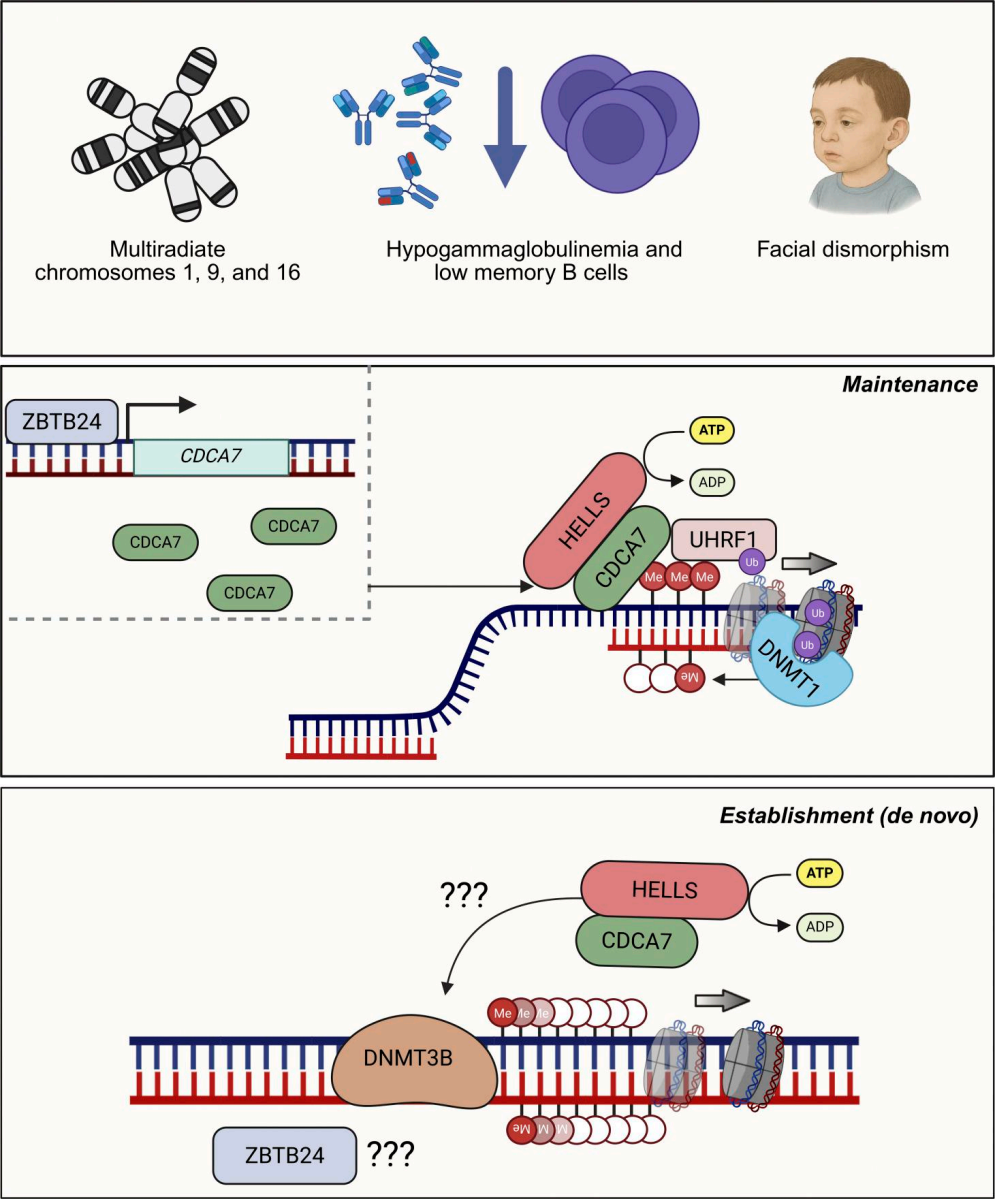
**Mechanisms responsible for hypomethylation in repetitive sequences in ICF syndrome.** All ICF patients are characterized by a multiradiate conformation of chromosomes 1, 9, and 16 due to hypomethylation of pericentromeric regions, immunodeficiency—mostly marked by hypogammaglobulinemia and reduced memory B cell levels—and specific facial anomalies. *DNMT3B*, mutated in the majority of patients (ICF1), mediates the de novo establishment of CpG methylation patterns. *ZBTB24*, *CDCA7*, and *HELLS* are additional genes in which causative mutations have been identified in ICF2, ICF3, and ICF4, respectively. These factors form a functional axis, consistent with the clustering of ICF2–4 subtypes. ZBTB24 acts as a transcriptional activator of CDCA7, which binds hemi-methylated CpGs during DNA replication and recruits HELLS, a chromatin-remodeling ATPase that promotes nucleosome sliding. This loosens heterochromatic regions and facilitates the ubiquitination of histone H3 by UHRF1, enabling DNMT1 binding and maintenance methylation during cell division. Although HELLS has been shown to directly interact with DNMT3B and participate in de novo methylation, it remains unclear whether and how the ZBTB24–CDCA7–HELLS axis contributes to this process.

ZBTB24’s role in ICF becomes clearer in the context of its interactions with CDCA7 and HELLS, aligning with a dichotomy between ICF1 and ICF2–4 patients. HELLS, a chromatin remodeling protein also known as lymphoid-specific (LSH), proliferation-associated SNF2-like, or SWI/SNF-related matrix-associated, actin-dependent regulator of chromatin group A6, is well known for its role in regulating genome-wide DNA methylation (89). This regulation is partly mediated through its interaction with UHRF1, facilitating chromatin association and H3 histone ubiquitination, thereby promoting DNMT1-mediated maintenance methylation (90). However, HELLS requires binding to CDCA7 to activate its ATPase activity, which enables nucleosome sliding and exposes CpG sites for methylation by DNMT1, assisted by UHRF1. In fact, CDCA7 provides binding specificity to preferentially hemimethylated pericentromeric regions during DNA replication (91, 92, 93). ZBTB24 enters this fold by directly binding to the *CDCA7* promoter and positively regulating its expression (73, 94) (Fig. 2). This ZBTB24–CDCA7–HELLS triad has also been shown to mediate methylation at the promoter region of the *Dux* gene cluster, whose aberrant expression retains mESCs in a totipotent state resembling the two-cell stage, potentially linking this mechanism to developmental defects observed in ICF patients (81).

Given the shared chromatin instability phenotype across all ICF subtypes, the ZBTB24–CDCA7–HELLS axis is also proposed to contribute to de novo methylation establishment. Indeed, HELLS has been shown to directly interact and recruit DNMT3B (95), as well as mediate transcriptional repression (95). Moreover, in mouse embryonic fibroblasts, knockout and subsequent re-expression of *Lsh* (HELLS) has been shown to restore de novo DNA methylation in a DNMT3B-dependent manner (96). Nevertheless, the mechanistic involvement of HELLS—and, consequently, CDCA7 and ZBTB4—in DNMT3B-mediated de novo DNA methylation requires further investigation (Fig. 2).

## From epigenetics alterations to immunodeficiency

A relevant question in ICF syndrome research is how alterations driven by genetic mutations in epigenetic and chromatin regulators are translated into immunodeficiency. Among ICF patients, a differential pattern is observed between DNMT3B mutants and nonmutants regarding the mechanisms underlying immune defects. In ICF1 patients, immunodeficiency is primarily thought to result from direct epigenetic disruption of B cell maturation—either directly or indirectly, as discussed above (97). Normal B cell differentiation—from hematopoietic progenitor cells to memory B and plasma cells—is accompanied by cumulative DNA methylation changes affecting ∼30% of all autosomal CpG sites. These changes primarily involve enhancer hypomethylation and the upregulation of key B cell transcription factors (e.g., BCL11A, EBF1, IRF4, MEF2A, MEF2C, PAX5, and TCF3), as well as increased methylation at polycomb-repressed regions associated with DNMT3A and DNMT3B activity (98). Genome-wide DNA methylation studies have consistently demonstrated the standard hypomethylation in pericentromeric heterochromatin but also changes in methylation in regulatory regions of genes involved in B cell immunity (86, 99). To date, the study by Gatto and collaborators is considered the most comprehensive exploration of epigenetics and transcriptomics in ICF1 lymphocytes. They analyzed patient-derived B-LCLs and demonstrated that DNMT3B defects lead to widespread genome-wide changes, including the expected loss of methylation and disruption of histone marks, as well as hypermethylation in a substantial number of regions. Moreover, hypomethylation of gene bodies promotes the acquisition of active histone marks (H3K4me3), enabling aberrant intragenic transcription start sites. A notable example is the expression of noncanonical isoforms of *PTPRC* (CD45), a key regulator of lymphocyte activation and development. Patient-derived cells also exhibited altered alternative splicing, correlated with gene body hypomethylation and reduced levels of H3K36me3, a mark of actively transcribed regions. ICF1 patients additionally showed increased expression of antisense transcripts, normally silenced by DNMT3B. A highlighted example is *CD27*-antisense, whose upregulation negatively correlated with the expression of *CD27*, a canonical marker of memory B cells that plays a key role in memory B cell differentiation (86). Finally, genomic instability resulting from hypomethylation may lead to increased apoptosis in rapidly dividing immune cells. During clonal expansion of B or T cells clonally in response to antigens, these cells may accumulate DNA damage, thereby limit immune repertoire formation and weaken immune responses.

In ICF2–4 patients, it is possible that some of these changes are due to general chromosomal instability. ICF2 patients share the hallmark phenotype of ICF1, namely centromeric instability, facial dysmorphism, and immunodeficiency, particularly memory B cell defects. Although both subtypes are clinically indistinguishable, some divergent patterns have been observed (Table 1). In ICF1 patients, immunodeficiency is generally more pronounced, while immunoglobulin class deficiencies are less severe in ICF2. ICF1 patients also tend to be diagnosed earlier due to the higher incidence of infections and slightly more severe hypogammaglobulinemia. Class-switch recombination (CSR) defects appear to be prevalent in ICF2 patients (100), while data on ICF1 patients are insufficient, as only an early study reported normal CSR in four ICF1 and non-ICF1 patients (6). Nevertheless, the more aggressive immunodeficiency in ICF1 patients may be associated with processes additional to CSR, which, in turn, appears to be mechanistically more linked to non-ICF1 patients, as we will explore below. Moreover, ICF2 shows a higher prevalence of intellectual disability (7, 49, 52). A gender bias has been proposed with a majority of ICF2 patients being male (32). From a molecular standpoint, hypomethylation of pericentromeric satellite II and III repeats is common to all ICF subtypes. However, ICF1 patients also exhibit subtelomeric hypomethylation accompanied by elevated transcription of telomeric repeat-containing RNA, due to a reduced promoter methylation. This is associated with accelerated telomere shortening and premature senescence (53). One can speculate whether this could be a cause of the higher severity of infections in ICF1, considering a comparable immunodeficiency phenotype (including low B cells) in telomere biology disorders such as dyskeratosis congenita. However, these disorders have a clearer T cell deficiency, which is lacking in ICF syndrome (101). In contrast, hypomethylation of α-satellite repeats is observed only in non-ICF1 patients (46, 52). In addition, differential methylation of specific regions—such as the promoters of germline genes *MAEL* and *SYCE1*, leading to their upregulation—has been reported in ICF1 but not ICF2 patients (34). The number of ICF3 and ICF4 cases remains too small to infer genotype-specific features for these subtypes; however, these patients tend to cluster with ICF2 patients (34, 53), supporting two main phenotypes: DNMT3B mutants (ICF1) versus DNMT3B nonmutants (ICF2–4). This is further corroborated by differential genome-wide DNA methylation patterns described in the blood of ICF patients. Although all ICF subtypes showed classical hypomethylation at pericentromeric repeats, differential profiles have been reported between ICF1 and ICF2-4. While mutations in *DNMT3B* show exclusive enrichment of hypomethylation in promoter CGIs (CpG islands) of germline genes, mutations in *ZBTB24*, *CDCA7*, and *HELLS* are particularly associated with low methylation of CpG-poor heterochromatin regions, mostly associated with monoallelic expressed genes involved in neuronal development (33).

**Table 1.** Summary of the non-concordant clinical and molecular features between ICF1 and ICF2–4 patients

​	ICF1	ICF2–4
Immunodeficiency	More pronounced and earlier diagnosis	Less extreme Ig class deficiencies
Intellectual disability	Lower prevalence	Higher prevalence
Gender bias	No significant bias noted	Majority male
Satellite DNA hypomethylation	Satellite II and III (common to all ICF) subtelomeric hypomethylation	Satellite II and III; α-satellite hypomethylation
Telomere biology	Elevated TERRA expression and telomere shortening	Not reported
Germline gene promoter methylation	Hypomethylation and upregulation of *MAEL* and *SYCE1*	Not reported
Genome-wide DNA methylation	Hypomethylation of promoters of germline genes	Hypomethylation of CpG-poor heterochromatin
CSR	Unexplored	Low IgG and IgA

TERRA, telomeric repeat-containing RNA.

As abovementioned, mutations in the ZBTB24–CDCA7–HELLS axis are particularly associated with defects in CSR in B lymphocytes. This impairment is linked to defective nonhomologous end-joining (NHEJ) during DNA repair. The roles of these three factors have been studied independently, with ZBTB24 investigated separately from CDCA7-HELLS. Isotype switching of immunoglobulins through CSR is a fundamental step in B cell maturation. Activation-induced cytidine deaminase (AID) induces DNA double-strand breaks at conserved motifs within switch (S) regions, located upstream of the coding regions of antibody heavy chains. These S regions are rejoined by NHEJ, leading to the removal of the μ and δ heavy chain constant regions, their substitution by a γ, α, or ε constant regions, and, consequently, a change in the class of immunoglobulins expressed by the B cell. Two mechanisms have been described for NHEJ. The classical pathway involves the DNA-dependent protein–kinase (DNA-PK) complex—comprising the KU70/KU80 heterodimer and the DNA-PK catalytic subunit—as well as the downstream effector proteins x-ray repair cross-complementing protein 4 (XRCC4), DNA ligase 4 (LIG4), and NHEJ factor 1. An alternative pathway can sustain NHEJ in the absence of the canonical mechanism. Although less well characterized, it has been shown to involve poly(ADP-ribose) polymerase 1 (PARP1), XRCC1, and DNA ligases 1 and 3 (102). In primary cells from ICF2 patients, Helfricht et al. observed impaired expression and release of IgG and IgA, which was attributed not to defects in the initiation of CSR, but rather to disruptions in its final steps, specifically in NHEJ. The proposed mechanism involves binding of ZBTB24 to poly(ADP-ribose) chains synthesized by PARP1, protecting PARP1 from degradation by poly(ADP-ribose) glycohydrolase, and facilitating recruitment of LIG4 and XRCC4 to mediate proper NHEJ. Notably, while PARP1 is primarily associated with alternative NHEJ, it has also been implicated in the classical pathway, as supported by these findings (100). Unoki and colleagues reported (in HEK293T and patient LCLs) that the CDCA7-HELLS axis, mediated by CDCA7, binds and recruits the KU70/KU80 (DNA-PK) complex, and that both components are essential for NHEJ (103). Furthermore, a comprehensive evaluation of the B cell phenotype in *Lsh* (HELLS) knockout mice revealed defects in CSR, not during the initiation, but in canonical NHEJ, resulting in impaired memory B cell maturation and reduced levels of all major IgG subclasses (104).

**Figure 3.**
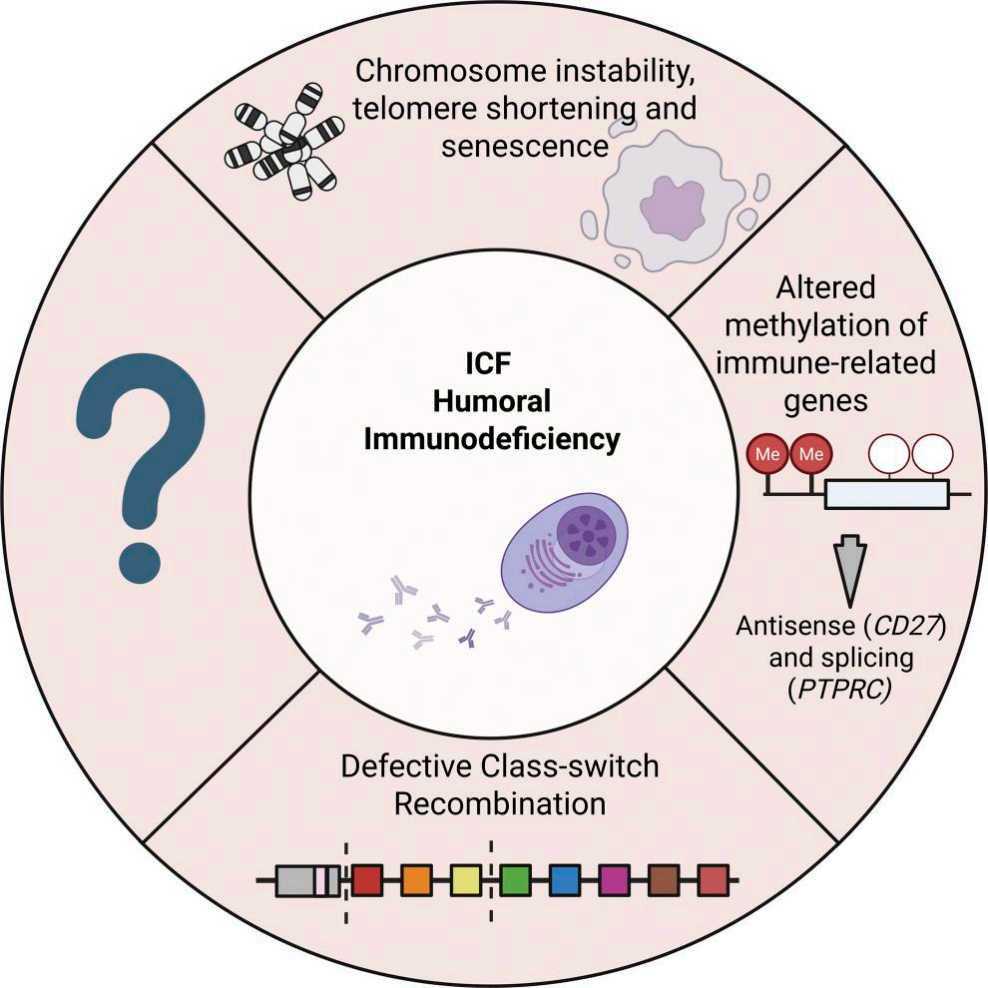
**Described causes for immunodeficiency in ICF.** Different mechanisms may be behind the development of humoral B cell immunodeficiency in ICF, some of them coincident and other distinct between subclasses of patients, and even between individual patients. Many of these, one must refer, are still to be properly described. Trans effects caused by chromosome instability caused by a genome-wide defective DNA methylation can translate in modifications to normal immune cell development, such as immune cell senescence caused by telomere shortening described in ICF patients (53). Such instability may lead to increased apoptosis in rapidly dividing immune cells, impairing clonal expansion of B (or T cells) in response to antigens, limiting the immune repertoire and weakening immune responses. Altered DNA methylation patterns of specific immune-related genes have also been described in ICF. The most prominent examples are *CD27*, whose promoter hypermethylation is associated with downregulation of the gene itself and upregulation of the antisense gene, and hypomethylation of intragenic regions of *PTPRC* (CD45) associated with modifications to exon splicing (86). Plus, inefficient CSR, particularly in ICF2–4 patients, appears to be an undisputed contributor (103, 104).

The cited studies provide detailed descriptions of key mechanisms associated with the immunodeficiency phenotypes observed in ICF patients. However, one must consider that the immunodeficiency in ICF may originate from different mechanisms, some of which are still unknown, and may vary among patients. These include direct hypomethylation and transcriptional dysregulation of immune genes, defects in CSR, trans effects from general hypomethylation, and chromosomal instability, such as telomere shortening and immune cell senescence, among others (Fig. 3). Ongoing investigation will be essential to further stratify ICF patients and to identify potential targets to treat the multiple levels of symptomatology in a personalized manner.

## ICF in the context of other epigenetic IEIs and future research

ICF can be compared to other IEIs that involve epigenetic dysregulation. For instance, Kabuki syndrome—caused by mutations in a histone MTase gene—often includes immunodeficiency (e.g., frequent infections and low Ig levels), reinforcing the importance of proper epigenetic marking in immune development (105). Similarly, an autosomal-recessive syndrome of immunodeficiency with lymphoproliferative disease has been associated with loss-of-function of ten-eleven translocation methylcytosine dioxygenase 2 (106). In addition, ataxia-telangiectasia (A-T)–like disorder (107) and Riddle syndrome, caused by mutations at the *RNF168* gene (108); A-T, caused by mutations at *ATM* (109); LIG4 syndrome, caused by mutations at *LIG4* (110); Nijmegen breakage syndrome caused by mutations at *NBN* (111); and Artemis deficiency, caused by mutations at *DCLREC1* (112), are examples of IEIs associated with defects in gene involved in double-strand DNA breaks and repair that also underscore the importance of genome maintenance in immunity.

Due to the scarcity of diagnosed ICF patients, our ability to fully characterize the mechanisms underlying the diverse clinical manifestations of the syndrome remains limited. One example is the lack of data on DNA methylation dynamics during B cell maturation in ICF. As extensively reviewed here, ICF patients clearly present with an immunodeficiency phenotype characterized by impaired B cell maturation, and defective DNA methylation has been observed in genes critical for this process (e.g., *CD27*). Nonetheless, this aspect has not been studied in as much detail as in other IEIs settings, and, as above mentioned, DNA methylation changes are paramount to B cell differentiation and proper function. Moreover, alterations in the DNA methylation profiles of B cells have been linked with chronic lymphocytic leukemia, where tumor cells are thought to originate from a continuum of B cell maturation states and accumulate aberrant epigenetic patterns (113). Nonetheless, we must be conservative in speculating on the extent to which these methylation changes directly drive the immunodeficiency phenotype.

Our group identified the occurrence of DNA methylation alterations in the transition from naïve to switched memory B cells in common variable immunodeficiency (CVID) (114), the most prevalent primary immunodeficiency, also marked by hypogammaglobulinemia. A comparison between CVID-discordant monozygotic twins revealed impaired demethylation during B cell differentiation, with differences most pronounced in switched-memory B cells. Notably, the regions that failed to demethylate in CVID corresponded to genes normally upregulated in germinal center B cells and were enriched for active enhancer histone marks (H3K4me1 and H3K27ac) (115). Single-cell RNA sequencing analysis further demonstrated that the transcriptional program of activated B cells in the CVID twin was also compromised and associated with the observed changes in DNA methylation, findings that were validated in a broader cohort of CVID patients (115). Although these studies were conducted on non-monogenic CVID patients, it is likely that epigenetic analysis of monogenic forms of primary antibody deficiencies within the CVID spectrum—affecting genes encoding different transcription factors and signaling pathways—will reveal both shared and gene-specific aberrant epigenetic profiles. In fact, DNA methylation profiling of B cells from patients with hyper-IgM syndrome type 2 (HIGM2)—a primary antibody deficiency caused by loss-of-function mutations in AID (which initiates CSR)—has revealed widespread alterations in DNA methylation and gene expression in naïve B cells. Of note, HIGM2 patients showed inability to properly demethylate during the naïve to memory B differentiation, and also an aberrant hypomethylation state in naïve B cells compared to controls. This suggests that AID loss affects DNA methylation dynamics in B cell differentiation and the establishment of the methylome in early stages of development. These changes have been interpreted as evidence of premature overstimulation of the B cell receptor prior to the germinal center reaction (116).

**Figure 4.**
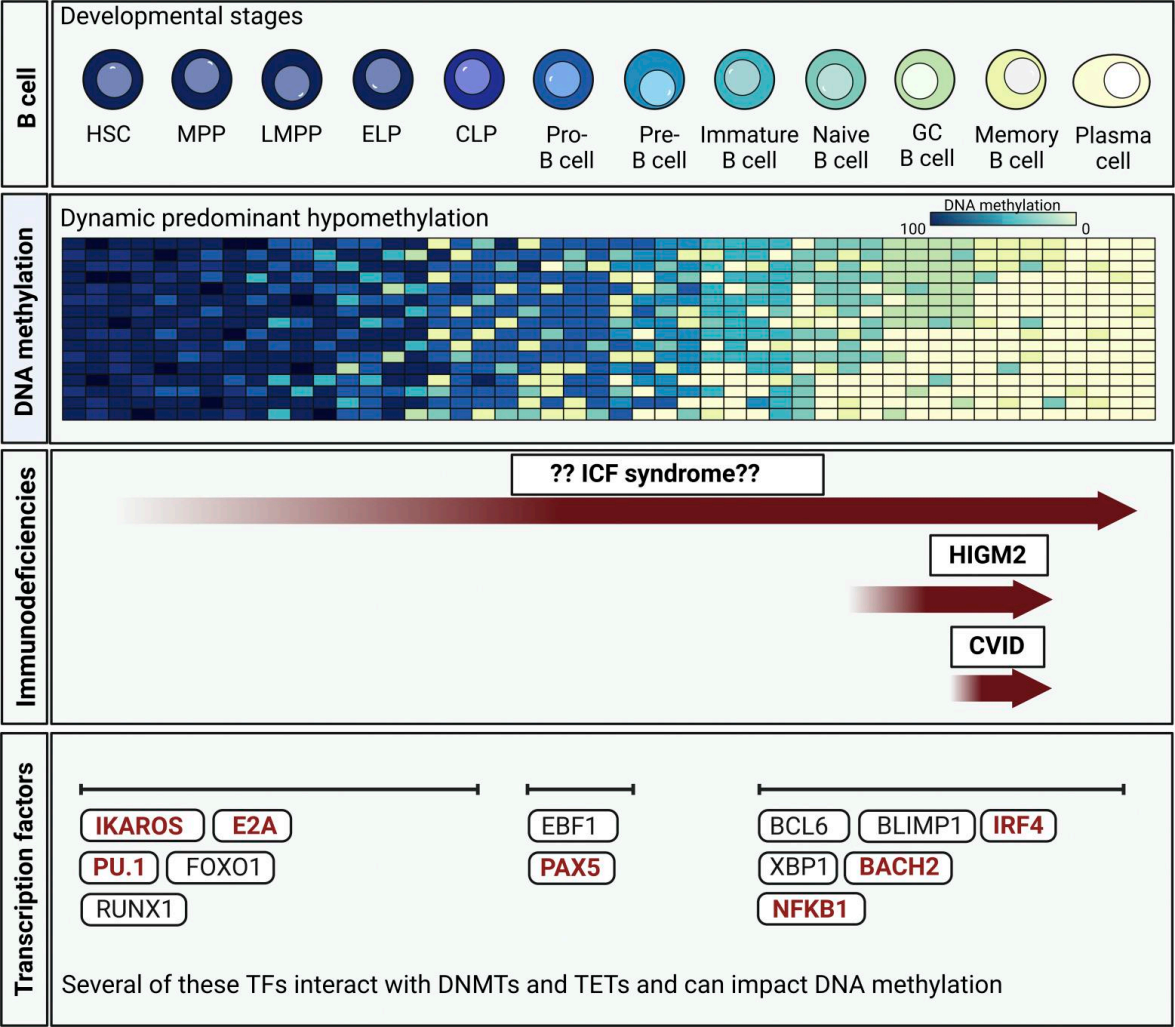
**ICF syndrome, along with other primary immunodeficiencies, in the context of DNA** **methylation changes occurring throughout the B cell differentiation (and activation) process.** Schematic representation of the previously described dynamic demethylation during B cell differentiation. Notably, although less prominent, methylation gains are also observed during this process (98, 113). In immunodeficiencies, defective DNA methylation changes have been reported in germinal center and memory B cells in CVID and HIGM2, with the latter also showing major changes at the naive B cell state (114, 115, 116). Despite the current lack of detailed understanding of DNA methylation modifications during B cell development, it is reasonable to speculate that, given the structural role of all four ICF-related genes in both the establishment and maintenance of DNA methylation patterns, ICF mutations could affect methylation dynamics at any stage of this process. Furthermore, transcription factors described to regulate the different steps of B cell differentiation, from early lineage commitment to peripheral activation and germinal center response, could be influencing or be influenced by altered DNA methylation landscapes and thus could guide in the understanding of epigenetic dysregulation in immunodeficiencies and represent targets for directed therapies (118). Some of these factors have, in fact, been shown to carry causative mutations in monogenic immunodeficiencies (indicated in bold red) (118). HSC, hematopoietic stem cells; MPP, multipotent progenitor; LMPP, lymphoid-prime multipotent progenitor; ELP, early lymphoid progenitor; CLP, common lymphoid progenitor; GC, germinal center.

Much is still to be understood regarding the axis proposed here, linking B cell differentiation, dynamic DNA methylation changes, immune-related transcription factors, and IEIs. For one, it is often difficult to demonstrate causality or correlation between DNA methylation changes and transcription. Moreover, transcription factors involved in B cell development and activation may either mediate DNA methylation modifications, through direct recruitment of enzymes implicated in introducing or removing DNA methylation, or have their binding and activation affected as a consequence of altered epigenetic landscapes. Of note, one should not disregard alterations to epigenetic profiles that can be a corollary of disordered B cell differentiation, regardless of the cause. Nonetheless, defects in dynamic DNA methylation changes during B cell development and activation have been shown in immunodeficiencies, such as CVID and HIGM2, as mentioned above, and causative IEI mutations have been described in several of these transcription factors—those involved in lineage commitment (IKZF1, E2A, SPI1, and PAX5) and in peripheral and germinal center activation (IRF4, BACH2, and NFKB1) (117). Due to its strong relationship with epigenetic and methylation defects, we propose that the continued study of ICF will potentially shed light on the axis between DNA methylation and B cell immunity, which is commonly disrupted across several immunodeficiencies (Fig. 4). Research on ICF syndrome illuminates fundamental connections between epigenetic regulation and the immune system, offering insights that extend to cancer biology—where DNA hypomethylation can drive genomic instability—and to other immunological diseases. Continued investigation may also reveal new epigenetic therapeutic targets that could benefit patients with ICF or related disorders in the future.

Current treatments like IVIG replacement—standard to manage antibody deficiency—and HSCT, performed in some ICF patients, can significantly improve immunoglobulin levels and infection outcomes. However, HSCT carries inherent risks and does not address nonimmune manifestations such as congenital dysmorphisms or increased cancer risk. Looking ahead, as we better understand the underlying epigenetic defects, one could envision therapies aimed at stabilizing the genome—for example, small molecules that enhance residual DNMT3B activity or compensate for HELLS/CDCA7 function—or gene therapy approaches to deliver corrected versions of the mutated genes to patient hematopoietic stem cells. While these remain theoretical at present, further research is strongly encouraged to one day bring them into clinical reality.

Taken together, findings in ICF underscore the central role of epigenetic regulation, particularly DNA methylation, in orchestrating proper B cell development and function—a process commonly disrupted in ICF and other IEIs. While current knowledge of ICF remains limited, findings from other immunodeficiency contexts raise the question of how deeper exploration of the B cell epigenome in a methylation-related syndrome like ICF—ultimately integrating the methylome, transcriptome, histone modifications, and chromatin architecture—could provide critical insights not only into disease pathogenesis but also into broader principles of immune regulation.
